# Overexpression of HACE1 in gastric cancer inhibits tumor aggressiveness by impeding cell proliferation and migration

**DOI:** 10.1002/cam4.1496

**Published:** 2018-04-19

**Authors:** Ying‐ling Chen, Dong‐ping Li, Hong‐yue Jiang, Yang Yang, Li‐li Xu, Shun‐cai Zhang, Hong Gao

**Affiliations:** ^1^ Department of Gastroenterology Zhongshan Hospital Fudan University Shanghai 200032 China; ^2^ Institute of Biochemistry and Cell Biology Shanghai Institutes for Biological Sciences Chinese Academy of Sciences Shanghai 200031 China; ^3^ Evidence‐Based Medicine Center of Fudan University Shanghai 200032 China

**Keywords:** Gastric cancer, HACE1, migration, proliferation, Wnt/*β*‐catenin pathway

## Abstract

HACE1 E3 ligase was discovered to be down‐regulated in several cancers while its role in regulating tumors was merely understood. This study aimed to explore the specific effect of HACE1 played in gastric tumorigenesis and its potential mechanism. HACE1's expression was found significantly lower in gastric cancer tissues compared with the adjacent normal tissues (*P* < 0.001). Its protein level in gastric cancer negatively correlated to tumor pathological differentiation (*P* = 0.019). And in gastric cancer patients with TNM I‐IIIa, those with lower HACE1 protein level had poorer overall survival (*P* = 0.025). Studies, in vivo and in vitro, showed that overexpressing HACE1 inhibited tumor proliferation and migration, and enhanced cell apoptosis. Besides, ectopic expression of HACE1 down‐regulated the protein level of *β*‐catenin and inhibited the activity of the Wnt/*β*‐catenin signaling pathway. All the cellular functions were abolished when we overexpressed inactive HACE1‐deltaHECT. Above all, we demonstrated that HACE1 E3 ligase played a suppressive role in gastric tumorigenesis and inhibited the activity of the Wnt/*β*‐catenin signaling pathway. Circumventing the decline of HACE1 in early stage of carcinoma may impede the tumorigenesis and malignant process of gastric cancer.

## Introduction

Gastric cancer, the fourth most common cancer worldwide and the second leading cause of cancer‐related death, has received great attention from many aspects 1. Although the adoption of endoscopy examination has mostly facilitated the earlier diagnosis of gastric cancer and timely surgery helps obtain a better prognosis, there are still thousands of patients dying with no effective strategy in place to reverse their advanced states. Therefore, continued concentration on the mechanism research of tumorigenesis and metastasis promises discoveries of new biomarkers and more innovative and effective treatments for patients with gastric cancer.

HECT domain and ankyrin repeat‐containing E3 ubiquitin protein Ligase (HACE1), one member of the HECT domain‐containing E3 ligase family, was first brought into our attention by the study of Wilms’ tumor 2 and was then frequently found lost or down‐regulated in many kinds of tumors 3, 4, 5, 6, 7, suggesting its role as a tumor suppressor. However, its special and pivotal role in tumor suppression was merely understood except for some discoveries suggesting that HACE1 could restrain reactive oxygen species generation 8, 9, control cell fate through regulating TNFR1 10, 11, and impede cell growth by accelerating ubiquitylation of Rac1 12. In previous work, we uncovered a special function of HACE1 as suppressing tumors by enhancing selective autophagy 13.

However, in gastric cancer, there is little exploration about the influence of HACE1 on tumorigenesis and metastasis. It is still unclear whether HACE1 plays a crucial role in gastric cancer proliferation or migration, or whether it predicts a better prognosis or is a potential biomarker which is in favor of innovative therapeutic approaches for gastric cancer.

In this study, we collected clinical samples and analyzed the correlation between HACE1 expression and overall survival as well as the clinicopathological features of the gastric cancer patients. Studies both in vitro and in vivo identified a suppressive role of HACE1 in impeding cell proliferation, migration, and inducing apoptosis through the down‐regulating Wnt/*β*‐catenin signaling pathway in gastric cancer.

## Materials and Methods

### Clinical specimens

The 142 pairs of clinical specimens including gastric cancer tissues and adjacent normal tissues were obtained from patients with gastric cancer who underwent gastrectomy, from 2006 to 2007, in Zhongshan Hospital, Fudan University, with the approval from the Research Ethics Committee of Zhongshan Hospital. And another six pairs of clinical tissues were collected in 2017. Informed consent was obtained from all participants included in the study. Detailed clinicopathological features of the 142 patients were collected and exhibited in Table 1.

**Table 1:** Correlation between HACE1 protein level in cancer tissues and the clinicopathologic features of the gastric cancer patients

Characteristics	Patients, *n*	Expression of HACE1		*p* value^b^
		High, *n* (%)	Low, *n* (%)	
Gender				
Male	100	15 (15.0)	85 (85.0)	0.385
Female	42	4 (9.5)	38 (90.5)	
Age				
≤55	57	5 (8.8)	52 (91.2)	0.189
>55	85	14 (16.5)	71 (83.5)	
TNM stages^a^				
I	17	3 (17.6)	14 (82.4)	0.802
II	38	4 (10.5)	34 (89.5)	
III	57	7 (12.3)	50 (87.7)	
IV	30	5 (16.7)	25 (83.3)	
Differentiation				
Low	91	7 (7.7)	84 (92.3)	0.019
High	51	12 (23.5)	39 (76.5)	

a UICC TNM classification.

b Spearman's correlation was performed to investigate the correlation between HACE1 expression and clinicopathological features of gastric cancer patients. Statistical significance was determined at the level of *p  *<* *0.05.

### Cell culture, plasmid construction, and infection

Six gastric cell lines (AGS, SGC‐7901, MKN‐45, MKN‐28, HGC‐27, and MGC803) were purchased from the cell bank of Chinese Academy of Sciences (Shanghai, China). MGC803 and were cultured in Dulbecco's modified Eagle's medium (Corning), and other cell lines were cultured in RPMI‐1640 Medium (Gibco, Nebraska, USA), supplemented with 10% fetal bovine serum (Gibco) at 37°C in a humidified atmosphere containing 5% CO_2_.

Lentivirus, pCDH‐HACE1‐EF1‐Puro, pCDH‐HACE1‐deltaHECT‐EF1‐Puro were designed and produced by the means described previously 13. After being infected by the lentivirus product, AGS and SGC‐7901 were cultured in a medium containing puromycin for selection of cell lines that stably expressed HACE1 or HACE1‐deltaHECT.

### CRISPR/Cas9 genome editing

HACE1 knockout in SGC7901 was achieved by means of CRISPR/cas9 genome editing assay. SgRNA targeting HACE1 was designed according to a gRNA designing tool from F. Zhang's laboratory (HACE1‐SgRNA‐F: CACCGCAACTCCACGGTGCGCGCG; HACE1‐SgRNA‐R: AAACCGCGCGCACCGTGGAGTTGC). Then, single vector carrying Cas9 nuclease (a gift from Ronggui Hu laboratory, Shanghai, China) and HACE1‐sgRNA was established and was transduced to SGC7901 by lentivirus. Special selection of SGC7901‐HACE1‐/‐ cell line was performed by adding puromycin, and then, individual clones were expanded in 48‐well plates. The protein level of HACE1 of each clone was detected by means of Western blot, and clones without HACE1‐detection were under DNA sequencing to confirm frameshifting indels.

### Immunohistochemistry

Tissues were fixed in formalin, embedded in paraffin, and sectioned before being mounted on slides which were then subjected to de‐paraffinizing and rehydrating. Then, the slides were microwaved for 30 min in 0.01 mol/L sodium citrate buffer (PH 6.0). After antigen retrieval and pre‐incubation with 10% normal goat serum, anti‐HACE1 (1:100; Proteintech, Chicago, USA) was employed at 4°C overnight. These slides were stained by the means of the VECTSDTSIN Elite ABC Kit (Vector Laboratories) and counterstained with hematoxylin. The intensity of staining was divided into four scales: 0 point, no staining; 1 point, weak, light yellow; 2 points, moderate, yellow‐brown; and 3 points, strong, brown. In addition, the proportion of positive cells was divided into four scales: 1, <25%; 2, 25%~50%; 3, 50%~75%; and 4, >75%. Then, the staining score was calculated by multiplying staining intensity with cell percentage. A staining score below 4 indicated low HACE1 expression while a score above 4 was considered high HACE1 expression.

### RNA extraction, reverse transcription, and real‐time RT‐PCR

Trizol (Invitrogen, Carlsbad, CA, USA) was used to extract total RNA of the six gastric cell lines and HEK293T, and then, reverse transcription was performed using Superscript II reverse transcriptase (Toyobo, Japan). Quantitative PCR amplification was finished using SYBR Green (Toyobo, Japan) on a CFX384 real‐time PCR machine (Bio‐Rad, Richmond, CA, USA). The primer of HACE1 for qPCR was produced by Boshang Biotech Company, Shanghai, China, and GAPDH which was used as normal control. The primer sequences of each gene were as follows: HACE1: 5′‐GAGAGAGCGATGGAGCAACT‐3′ and 5′‐ACAGCAAAACCAAGCATTCC‐3′; GAPDH: 5′‐GAGTCAACGGATTTGGTCGT‐3′ and 5′‐TGGAAGATGGTGATGGGATT‐3′.

### Cell proliferation assay and colony formation assay

Cells were plated in 96‐well plates at 4000 cells per well, and CCK‐8 (Beyotime Biotechnology, Shanghai, China) was used to detect the cell viability at 450 nm after incubation for half an hour. Proliferation of cells was determined by adding CCK‐8 for detection at 0, 24, 48, 72 h, separately. For colony formation assay, cells were plated in 6‐well plates at 400 cells per well. Then, the cell colonies, formed after incubating 7–12 days, were fixed by 4% paraformaldehyde, stained by 0.5% crystal violet, and measured by detecting at 595 nm.

### Wound‐healing assay

Cells were plated into 6‐well plates at 1 × 10^5^ cells per well, and 200‐mL tips were used to scratch the monolayer of cells when they grew to 75% confluence in each well. Then, the plates were washed by PBS for twice and filled with a serum‐free medium for 24 h. Images of cells migrating at the wound sites were captured by an inverted microscope, and the rate of wound healing was calculated by the formula: the rate of wound healing = (the wound width of 24 h/ wound width of 0 h) × 100%.

### Transwell migration assay

The Transwell chambers (8 mm pores; Corning) were prepared with incubation in Matrigel (BD Biosciences, New Jersey, USA). Cells were digested by trypsin (Gibco) and suspended with a RPMI‐1640 medium containing 1% serum. After counting and calculating the concentration of cells, 8 × 10^3^ cells were transferred to the upper chambers, while the lower chambers were filled with a 400 μL medium containing 10% serum. Then, the cells migrating to the lower chamber were fixed by 4% paraformaldehyde, stained by 0.5% crystal violet, and counted by an inverted microscope.

### Western blotting

Total proteins were extracted from cells infected or uninfected by lentivirus, and their concentrations were measured by means of a BCA protein assay kit (Beyotime, Shanghai, China). Then, cell lysates were loaded on SDS‐PAGE for separation, transferred onto the PVDF membrane, and blocked with 5% milk. After incubation with relevant primary antibodies at 4°C overnight, the membrane was incubated with species‐matched secondary antibodies. The antibodies used exhibited as follows: HACE1 (1:500; Abcan 133637), caspase‐9 (1:1000; Bioworld Technology, St. Louis Park, MN, USA), caspase‐8 (1:1000; Bioworld Technology), caspase‐3 (1:1000; Bioworld Technology), *β*‐catenin (1:1000; Bioworld Technology), and GAPDH (1:2000; Santa Cruz, CA, USA). The expression level of proteins was detected by means of an enhanced chemiluminescence detection kit (Thermo Scientific, Waltham, MA, USA).

### TopFlash assay

Cells were prepared in 24‐well plates, and were cotransfected with TopFlash reporter plasmids (300 ng, a gift from Hu laboratory) and pTK‐RL plasmids (100 ng) by the means of Lipofectamine 2000 (Invitrogen, Carlsbad, CA, USA). After 24 hours, Wnt agonist I (Selleck, Houston, Texas, USA) was added to stimulate the Wnt/*β*‐catenin signaling pathway. The activity of firefly and Renilla luciferase reporters was determined 24 hours after induce using the Dual Luciferase Assay kit (Promega, Madison, Wisconsin, USA). The values were normalized to Renilla luciferase.

### Tumor xenograft assay

Four‐week‐old athymic female nude mice were purchased from Shanghai SLAC Laboratory Animal Co., Ltd (Shanghai, China). All animal studies were performed under the approval from the Animal Care and Use Committee of Zhongshan hospital, Fudan university. SGC7901 cells with or without ectopic expression of HACE1 were injected subcutaneously (1 × 10^7^ cells per mouse) into the mice, respectively. After 21 days, the tumors were measured using a caliper and their weights were measured, too.

### Statistical analysis

All data were analyzed using GraphPad prism 6.0 software (CA, USA). Experimental data implicated contained at least three replicates and were presented as the mean ± standard deviation. Differences between groups were analyzed by Student's *t*‐test, and the proliferation curves were analyzed by two‐way ANOVA. The survival curves were calculated by the means of a Kaplan–Meier analysis, and the correlation between HACE1 expression and clinicopathological features of gastric cancer patients was analyzed using Spearman's rank correlation analysis which was followed by a lineal correlation analysis when the Spearman turned out to be significant. Statistical significance was determined at the level of *p *<* *0.05.

## Results

### Low expression of HACE1 in gastric cancer tissues and cell lines

**Figure 1:**
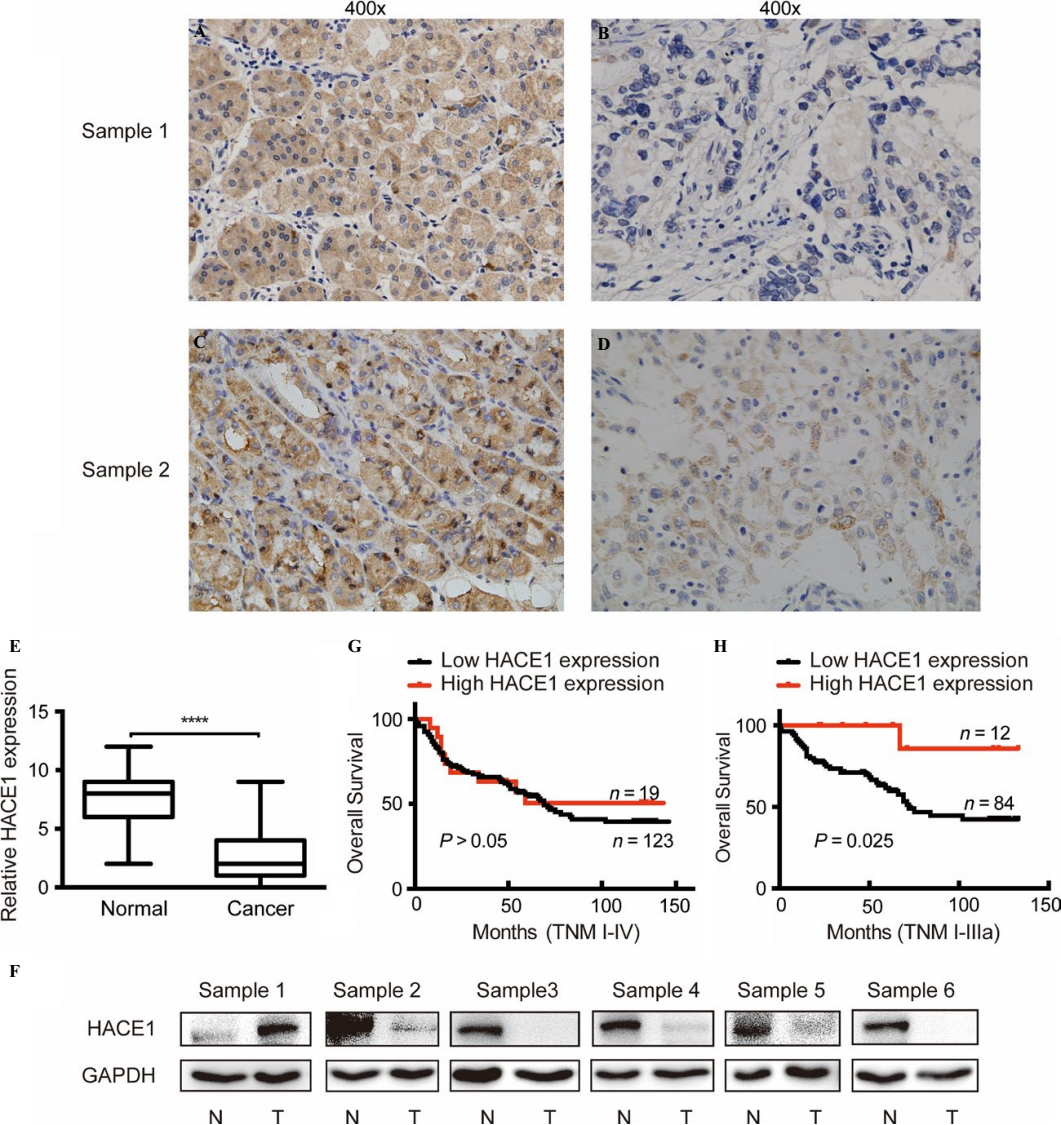
HACE1 expression levels in clinical gastric cancer tissues and adjacent normal tissues, and its correlation to survival of patients with gastric cancer. (A, B) Images of immunohistochemical (IHC) detection of HACE1 expression in sample 1. (A) Adjacent normal tissues: strong staining (400×). (B) Cancer tissues: light staining (400×). (C, D) Immunohistochemical detection of HACE1 expression in sample 2. (C) Adjacent normal tissues: strong staining (400×). (D) Cancer tissues: medium staining (400×). (E) Protein levels of HACE1 measured by IHC. IHC scores of HACE1 were calculated as the staining intensity (0, 1, 2, or 3) multiplied by the staining extent (0–100%). (F) The Western blot of HACE1 protein level in different pair of clinical gastric tissues. N: normal; T: tumor. (G) Kaplan–Meier curve analysis of HACE1 expression levels with overall survival time of 142 patients with gastric cancer by the log‐rank test. (H) Kaplan–Meier curve analysis of HACE1 expression levels with overall survival time of gastric cancer patients with TNM ranging from I to IIIa stages (****P <* *0.0001).

**Figure 2:**
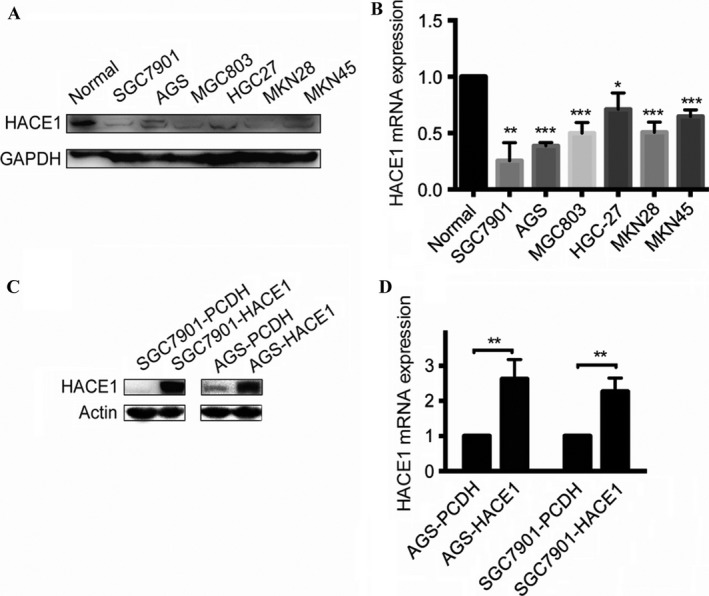
The expression level of HACE1 in gastric cancer cell lines. (A) Detection of HACE1 protein in six gastric cancer cell lines compared with human normal gastric tissues by means of Western blot. (B) The mRNA levels of HACE1 in gastric cancer cell lines and human normal gastric tissues were calculated by qRT‐PCR. (C) The HACE1 protein levels in gastric cancer cell lines with or without infection of lentivirus carrying HACE1. (D) The mRNA levels of HACE1 in gastric cancer cell lines with or without infection of lentivirus carrying HACE1. All experiments were conducted in triplicates. Error bars, SD of the mean and statistical comparisons were performed using unpaired *t*‐tests (*p <* *0.05; **p <* *0.01; ***p <* *0.001).

To explore the expression of HACE1 in gastric cancer tissues, we studied 142 pairs of clinical samples of patients with gastric cancer by the means of immunohistochemistry (IHC) and observed a distinctly decreased expression of HACE1 in cancer tissues compared with the adjacent normal tissues (Fig. 1A–E). The Western blot also showed a down‐regulation of HACE1 in human gastric cancer tissues as compared to the adjacent normal tissues (Fig. 1F). Besides, we found that six gastric cancer cell lines exhibited low mRNA and protein level of HACE1 when compared to human normal gastric tissues (Fig. 2A and B). These data implied a potential tumor suppressive role of HACE1 in gastric cancer.

### Correlations between HACE1 expression and clinicopathological features in gastric cancer patients

As HACE1 might play a suppressive role in gastric cancer, we tended to evaluate the correlation of different amounts of HACE1 expressed in cancer tissues to overall survival of these patients. It revealed that there was no significant difference in patients with or without high level of HACE1 (Fig. 1F). However, among patients with TNM ranging from I to IIIa stages, those with higher levels of HACE1 obtained a longer survival time after surgery (*p *=* *0.025; Fig. 1G). Moreover, Spearman rank correlation analysis revealed that low expression of HACE1 in tumors was closely correlated with poor pathological differentiation (*p *=* *0.019), yet it had no relationship with TNM stage, patient age and gender (Table 1). Therefore, HACE1 might function as a differentiation enhancer and a potential prognostic factor.

### HACE1 inhibits gastric cancer cell proliferation in vitro

**Figure 3:**
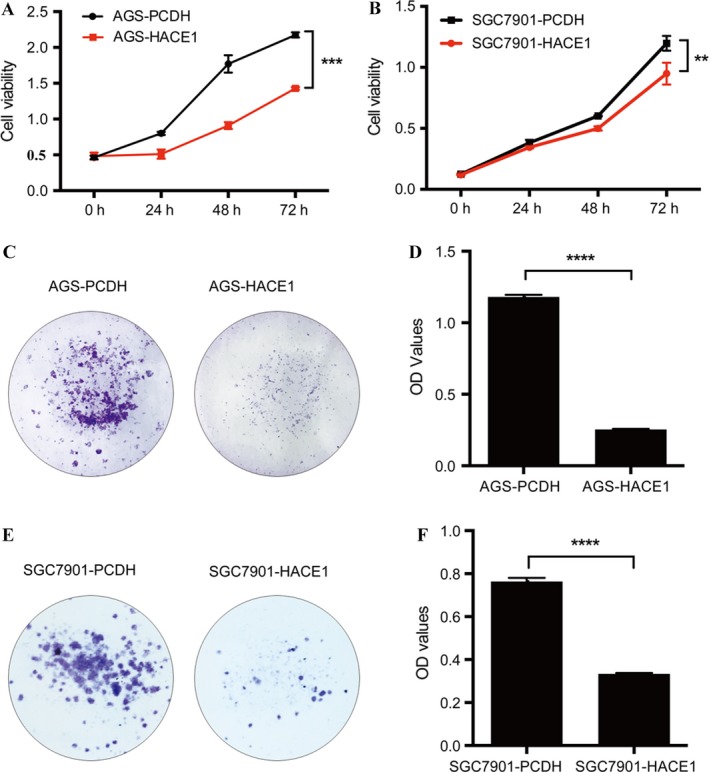
The influence of HACE1 expression on cell proliferation of gastric cancer cell lines. (A, B) Effect of overexpression of HACE1 in AGS and SGC7901 on cell growth. The number of cells was measured in samples at 0, 24, 48, and 72 h separately by means of CCK‐8 which was detected under 450 nm. (C, E) The pictures of colonies of AGS and SGC7901 with or without overexpression of HACE1. Cells were stained with 0.2% crystal violet. The OD values (D, F) were detected at 589 nm after dissolved by 3% ethylic acid. All experiments were replicated by three times. Error bars, SD of the mean and statistical comparisons were performed using Student's *t*‐tests (**p <* *0.01; ***p <* *0.001; ****p <* *0.0001).

As substantially low expression of HACE1 was found in several gastric cancer cell lines (Fig. 2A and B), we established two cell lines stably expressing ectopic HACE1, AGS‐HACE1 and SGC7901‐HACE1, by introducing lentivirus carrying the HACE1 gene (Fig. 2C and D). It revealed that the viability of cells overexpressing HACE1 was lower than that of the control groups, AGS‐PCDH and SGC7901‐PCDH, at every 24, 48 and 72 h (Fig. 3A and B). Besides, the number of cell colonies in control cell lines was significantly larger compared with the HACE1‐overexpression groups (Fig. 3C–F). Therefore, these data showed that elevation of HACE1 could remarkably block cell proliferation and colony formation.

### HACE1 inhibits gastric cancer cell migration in vitro

**Figure 4:**
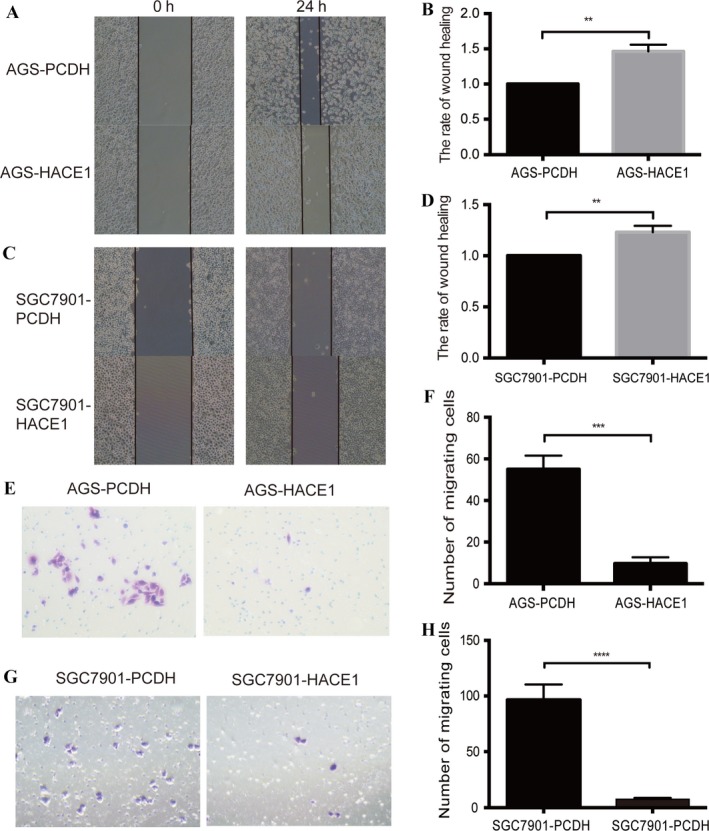
Impact of HACE1 expression on cell migration of gastric cancer cell lines in vitro. (A, C) Impact of HACE1 overexpression on cell migration by means of wound‐healing assay in AGS and SGC7901. The pictures were taken at 0 and 24 h, respectively (200×). (B, D) The rates of the width of each scratch. The rate of wound healing = (the wound width of 24 h/ wound width of 0 h) × 100%. (E, G) The images of migrating cells of AGS and SGC7901 were taken after infecting the samples with lentivirus carrying HACE1 (400×). After 24 h in Transwell assay, the number of cells migrating to the lower chamber was calculated and analyzed separately (F, H). All experiments were performed in triplicates. Error bars, SD of the mean and statistical comparisons were performed using Student's *t*‐tests (**p <* *0.01; ***p <* *0.001; ****p <* *0.0001).

We also explored the effect of HACE1 on cell migration. Wound‐healing assay showed that cell groups overexpressing HACE1 kept longer widths 24 h after scratching as compared to the control groups which exhibited much smaller widths (Fig. 4A–D), implying a poorer migration ability of cells with an elevated level of HACE1. Besides, there were more cells in the SGC7901‐PCDH and AGS‐PCDH groups migrating to the lower chambers compared to the cell groups overexpressing HACE1, respectively (Fig. 4E–H). The results of a poorer migration ability and a lower migrating rates in HACE1‐overexpression cells, exhibited by wound‐healing and Transwell assays, demonstrated a suppressive role of HACE1 on cell migration in gastric cancer.

### HACE1 induces gastric cancer cell apoptosis

**Figure 5:**
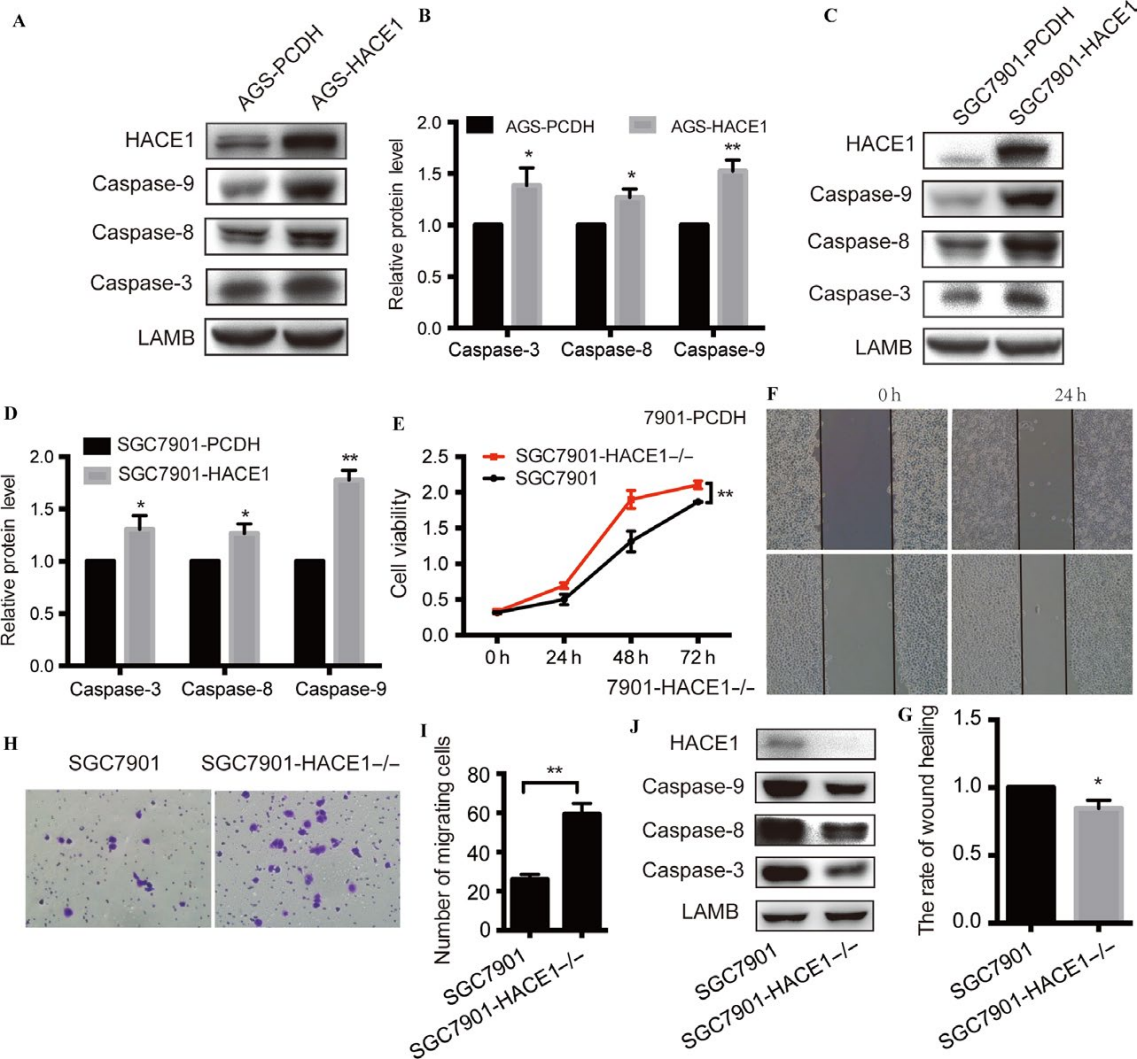
HACE1 induces cell apoptosis and the effect of HACE1 deletion in gastric cancer cell lines. (A–D) Overexpression of HACE1 in AGS and SGC7901 elevated caspase‐9, caspase‐8, and caspase‐3 protein levels. (E) Effect of HACE1 deletion in SGC7901 on cell growth. The number of cells was measured in samples at 0, 24, 48, and 72 h separately by means of CCK‐8 which was detected under 450 nm. (F) Pictures of HACE1 knockout on cell migration by means of wound‐healing assay in SGC7901. The pictures were taken at 0 and 24 h, respectively (200×). (G) The rate of the width of each scratch. The rate of wound healing = (the wound width of 24 h/ wound width of 0 h) × 100%. (H) The images of migrating cells of SGC7901 with HACE1 knocked out were taken after 24 h in Transwell assay. (I) The number of SGC7901 cells migrating to the lower chamber. (J) Protein levels of caspase‐9, caspase‐8, and caspase‐3 in SGC7901 cells with or without HACE1 deletion. (*p <* *0.05; **p <* *0.01).

The effect of HACE1 on cell apoptosis was investigated. Evidence showed that the amount of caspase‐9 protein was remarkably increased after overexpressing HACE1 in AGS, and its subsequent target caspase‐3 and another apoptotic molecule, caspase‐8, were also noted as having relative elevation compared with the control group (Fig. 5A and B). The SGC7901 cell lines showed a similar increase in caspase‐9, caspase‐3, and caspase‐8 proteins when elevating the protein level of HACE1 (Fig. 5C and D). These data suggested that HACE1 could enhance programmed cell death in gastric cancer.

### HACE1 knockout promotes tumor proliferation and migration

To verify the influence of HACE1 on gastric cancer, we knocked out HACE1 in SGC7901 cells and replied the experiments above. It revealed that deletion of HACE1 increased cell growing rate when compared with the SGC7901‐control cell line (Fig. 5E). Besides, cells without HACE1 expression moved faster than the control group (Fig. 5F and G), and there were more cells transferring into the lower chamber according to the Transwell assay in HACE1 knockout group (Fig. 5H and I). In the protein level, HACE1 deficiency inhibited the protein expression of caspase‐3, caspase‐8, and caspase‐8 (Fig. 5J), indicating a suppression on cell apoptosis.

### HACE1 loses its function on gastric cancer when deprived of its E3 ligase activity

**Figure 6:**
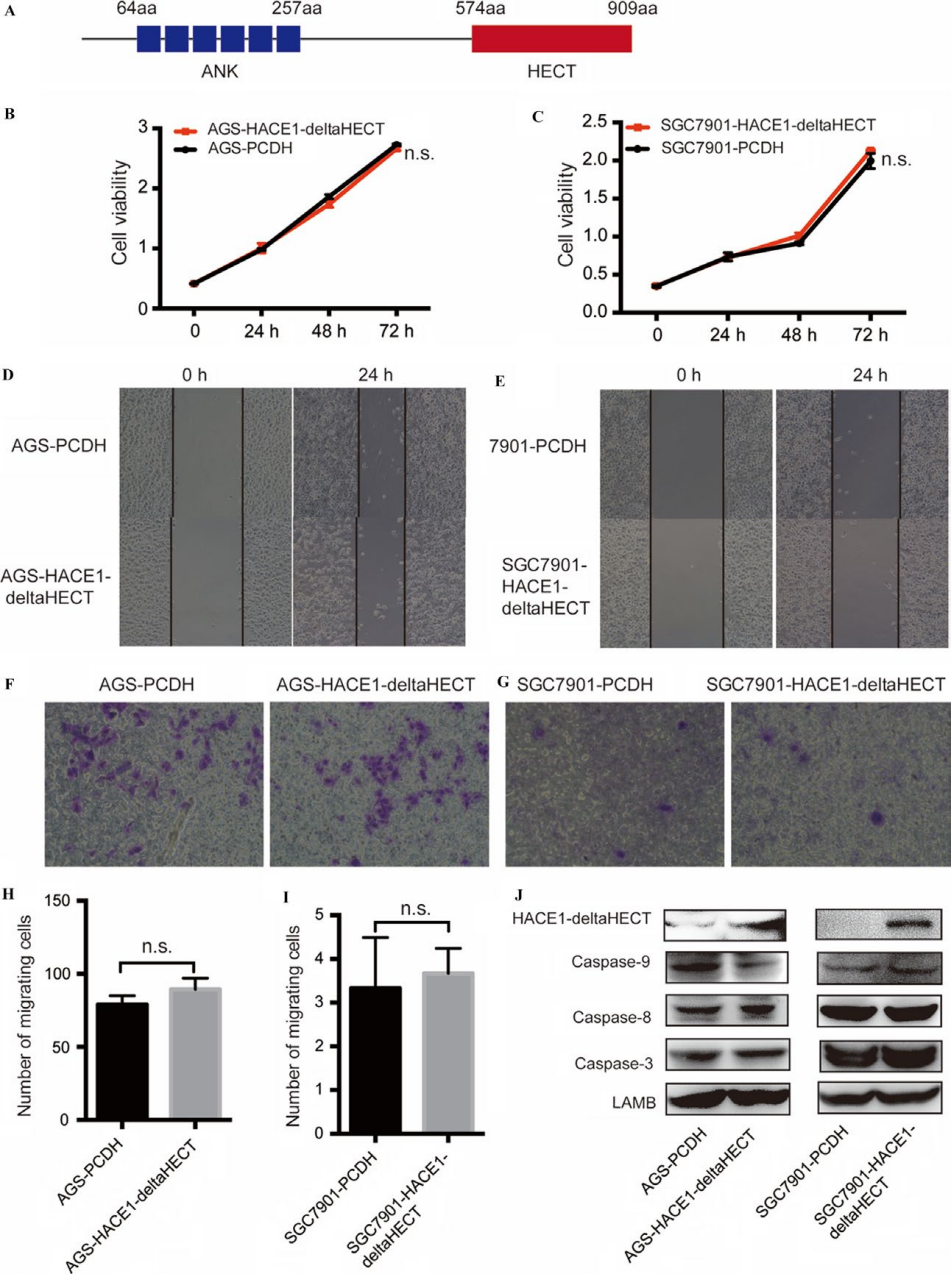
Effect of inactive HACE1 on gastric cell lines. (A) Picture of HACE1's structure. “aa”: amino acid. (B, C) Effect of HACE1 HECT domain deletion on cell growth in AGS and SGC7901. The number of cells was measured in samples at 0, 24, 48, and 72 h separately by means of CCK‐ 8 which was detected under 450 nm. (D, E) Pictures of HACE1‐deltaHECT on cell migration by means of wound‐healing assay in AGS and SGC7901. The pictures were taken at 0 and 24 h, respectively (200×). (F, G) The images of migrating cells with or without HACE1‐deltaHECT were taken after 24 h in Transwell assay. (H, I) The number of cells migrating to the lower chamber. (J) Protein levels of caspase‐9, caspase‐8, and caspase‐3 in SGC7901 cells with or without HACE1 deletion (n.s.: not significant for the indicated comparison).

HACE1, one member of the HECT domain‐containing E3 ligase family, consists of six Ankyrin repeats at its N‐terminal and HECT domain at its C‐terminal which catalyzes the binding of ubiquitin to substrate proteins 2, 14 (Fig. 6A). Deleting HECT domain makes HACE1 inactive to target substrates for ubiquitylation. To confirm whether HACE1 executes its function in gastric cancer through E3 ligase activity, we established a HACE1 inactive mutant with the HECT domain deleted, HACE1‐deltaHECT, and then performed CCK‐8 and Transwell assays to examine the cell proliferation and migration abilities of the HACE1 inactive mutant cell lines. The results showed that overexpressing HACE1‐deltaHECT had no significant effect on cell proliferation (Fig. 6B and C) or migration (Fig. 6D–I) in AGS and SGC7901 cells. The apoptotic proteins showed similar expression levels in HACE1‐deltaHECT and the control group of PCDH (Fig. 6J). Altogether, these results showed that HACE1 has mere regulation on gastric cancer cell lines when deprived of its E3 ligase activity.

### HACE1 inhibits the Wnt/*β*‐catenin signaling pathway

**Figure 7:**
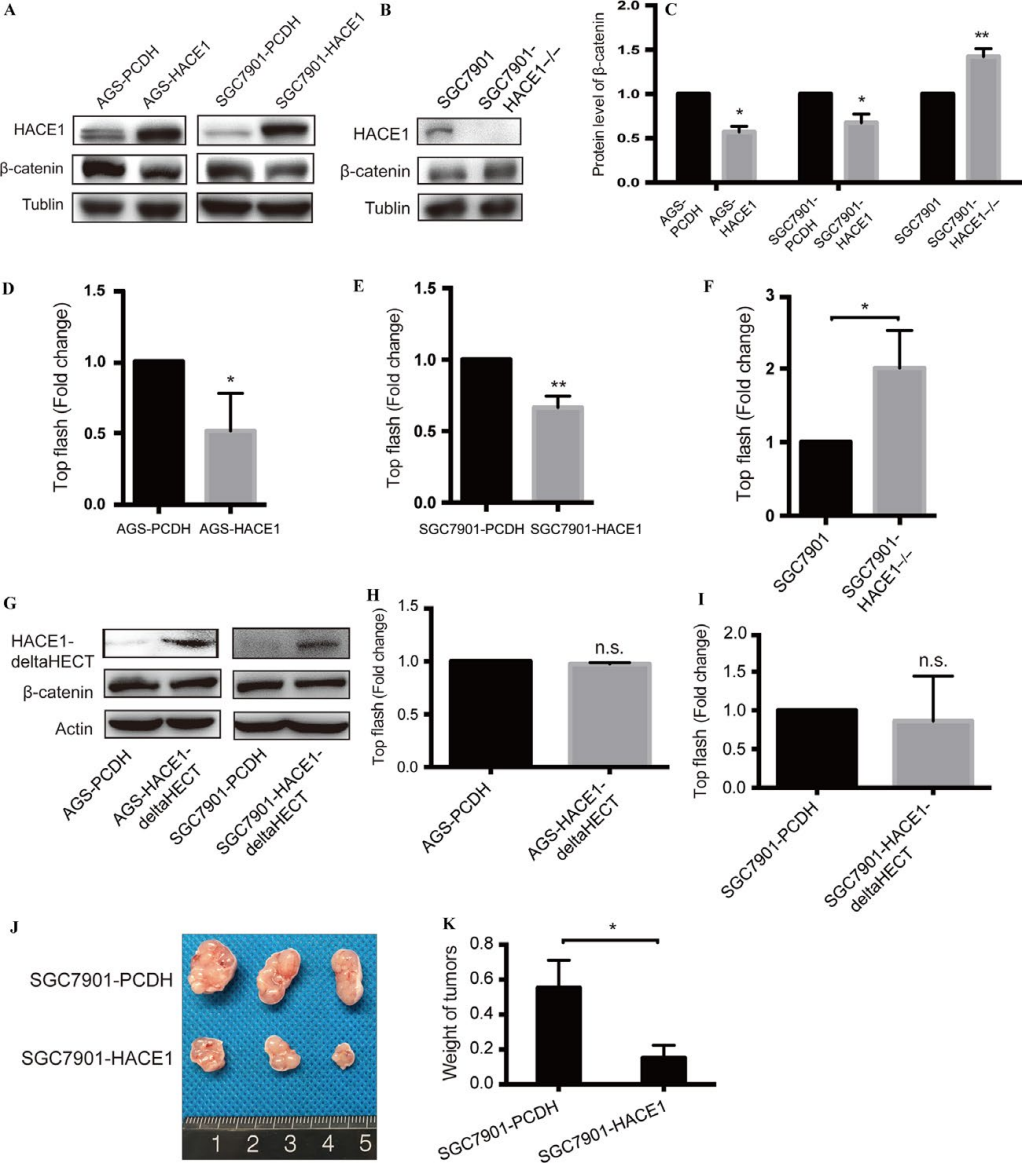
HACE1 down‐regulates the Wnt/*β*‐catenin signaling pathway. (A, C) The protein level of *β*‐catenin in AGS and SGC7901 with or without HACE1 overexpression. (B, C) The protein level of *β*‐catenin in SGC7901 with HACE1 knockout. (D, E) The activity of the Wnt/*β*‐catenin signaling pathway by TopFlash assay in AGS and SGC7901 with or without HACE1 overexpression. (F) The activity of the Wnt/*β*‐catenin signaling pathway by TopFlash assay in SGC7901 with HACE1 knockout. (G) Influence on *β*‐catenin protein level by HACE1 HECT domain deletion. (H, I) The activity of the Wnt/*β*‐catenin signaling pathway by TopFlash assay in AGS and SGC7901 with or without HACE1‐deltaHECT. (J) The picture of xenograft tumors of nude mice (21 days). (K) Overexpression of HACE1 reduced the weight of xenograft tumors of nude mice (21 days) (*p <* *0.05; **p <* *0.01; n.s.: not significant for the indicated comparison).

The Wnt/*β*‐catenin signaling pathway played a crucial role in promoting cell proliferation and migration 15, 16, and previous studies revealed that HECT domain‐containing E3 ligases were implicated in down‐regulation of Wnt/*β*‐catenin signaling 17, 18. As one member of the HECT domain‐containing E3 ligases family, we postulated that HACE1 might inhibit cell proliferation and migration through down‐regulation of the Wnt/*β*‐catenin signaling pathway. Intriguingly, overexpressing HACE1 in AGS and SGC7901 reduced *β*‐catenin protein level (Fig. 7A and C) and inhibited the activity of the Wnt/*β*‐catenin signaling pathway (Fig. 7D and E), while knocking out HACE1 could increase the protein level of *β*‐catenin (Fig. 7B and C) and enhance the activity of the Wnt/*β*‐catenin signaling pathway (Fig. 7F). To identify whether HACE1 executed its regulation of the Wnt/*β*‐catenin signaling pathway through its ligase activity, we detected the change in *β*‐catenin protein and its activity when deleting the HECT domain. The Western blot presented no significant change in *β*‐catenin protein under HECT deletion (Fig. 7G). There was no significant change in the activity of the Wnt/*β*‐catenin signaling pathway (Fig. 7H and I), either, when overexpressing inactive HACE1‐deltaHECT. These results revealed that HACE1 could regulate the Wnt/*β*‐catenin signaling pathway and inhibit its activity through its E3 ligase function in gastric cancer.

### HACE1 inhibits tumor growth in vivo

To testify the influence of HACE1 on tumor growth, we performed in vivo study by injecting SGC7901 cells subcutaneously into nude mice. It showed that the tumors of mice injected with SGC7901‐HACE1 cells turned out to be much smaller in size than that of control groups which were injected with SGC7901‐PCDH cells (Fig. 7J). Besides the weight of subcutaneous tumors in HACE1 overexpression group was lower than that in the control group (HACE1 vs. PCDH, 0.1517 ± 0.0417 vs. 0.5524 ± 0.0918 g, *p *=* *0.0165; Fig. 7K). Taken together, this study demonstrated that HACE1 overexpression was able to suppress the tumor growth of gastric cancer in vivo.

## Discussion

HACE1 was found low expressed in previous studies about breast cancer 3, colorectal cancer 5, liver cancer 7 and leukemia 19. In this study, we also spotted a significant down‐regulation of HACE1 in gastric cancer tissues compared with their adjacent normal tissues.

Intriguingly, the aberrant low expression of HACE1 notably correlated to a poor pathological differentiation of tumor. As poor pathological differentiation renders tumor to grow faster and be more likely to develop invasive phenotype, this correlation might provide an indication that HACE1's abnormal depletion was related to tumorigenesis and malignant appearance of gastric cancer. Besides, the survival data revealed that the patients within IIIa stages who carried high level of HACE1 exhibited a better prognosis, from which we assumed that HACE1 was beneficial for patients in early stage and blocking the abnormal down‐regulation of HACE1 in early stage might give an inspiration to enhance the treatment of patients with gastric cancer. Intriguingly, several studies have revealed HACE1 was epigenetically inactive by gene methylation on its promoter in hepatocellular carcinoma 20 and colorectal cancer 5, as well as gastric cancer 21. The epigenetic disorder‐induced down‐regulation of HACE1 facilitated the tumorigenesis of gastric cancer; thus, blocking the aberrant epigenetic regulation might restore the expression of HACE1, which is a promising approach worthy of further studies.

Apart from the discovery of a low expression level in clinical gastric cancer tissues, we also found a concordant low level of HACE1 in six gastric cancer cell lines as compared to human normal gastric tissues. Consistently, two of these cell lines with forced overexpression of HACE1 exhibited limited ability of proliferation as well as migration, and at the same time, they were more subject to cell apoptosis. On the contrary, knocking out HACE1 facilitated the cell growth and migration. And in vivo, the tumor xenograft study provided a further exhibition of HACE1's function as inhibiting the growth of gastric tumor in nude mice. These studies significantly demonstrated that HACE1 exerted a suppressive influence on gastric cancer.

The Wnt/*β*‐catenin signaling pathway, according to previous studies, could promote cancer cell proliferation and migration 22, 23 and be down‐regulated by several members of HECT domain‐containing E3 ligases like Huwe1 24. Given these studies, we assume that HACE1, one member of the HECT family, might also target the Wnt pathway to regulate cell proliferation and migration. To figure out this question, first we established an inactive HACE1 with its HECT domain deleted. This inactive HACE1‐deltaHECT lost its suppressive regulation on gastric cancer proliferation and migration, indicating that HACE1 exerted its influence on gastric cancer through its E3 ligase activity. Furthermore, when elevating the expression of wide‐type HACE1, we could see a down‐regulation of *β*‐catenin protein, and depression of the Wnt/*β*‐catenin pathway activity, while HACE1‐deltaHECT did not inhibited the protein level of *β*‐catenin nor its pathway activity.

Intriguingly, these results, in accordance with our hypothesis, strongly indicate that HACE1 might inhibit the Wnt/*β*‐catenin signaling pathway. However, whether there is a pivotal substrate in the Wnt/*β*‐catenin signaling pathway that could be targeted by HACE1 for ubiquitylation and degradation is unknown, and it deserves further exploration.

In all, our studies unveiled a suppressive role of HACE1 in tumor growth and migration of gastric cancer, and it might help to provide novel insights into the blockage of tumorigenesis and malignant process of early stage of gastric cancer.

## Conflict of Interest

The authors declare that they have no conflict of interest.

## Supporting information


**Appendix S1.** The effect of HACE1 mutation on gastric cancer cell lines.
