# Supplementary material for: Overexpression of HACE1 in gastric cancer inhibits tumor aggressiveness by impeding cell proliferation and migration
Source: Cancer Med. 2018 Apr 19;7(6):2472–84. doi: 10.1002/cam4.1496 (PMC6010910; doi:10.1002/cam4.1496)
Supplement: Supplementary file 1 — Appendix S1. The effect of HACE1 mutation on gastric cancer cell lines. [file CAM4-7-2472-s001.pdf]

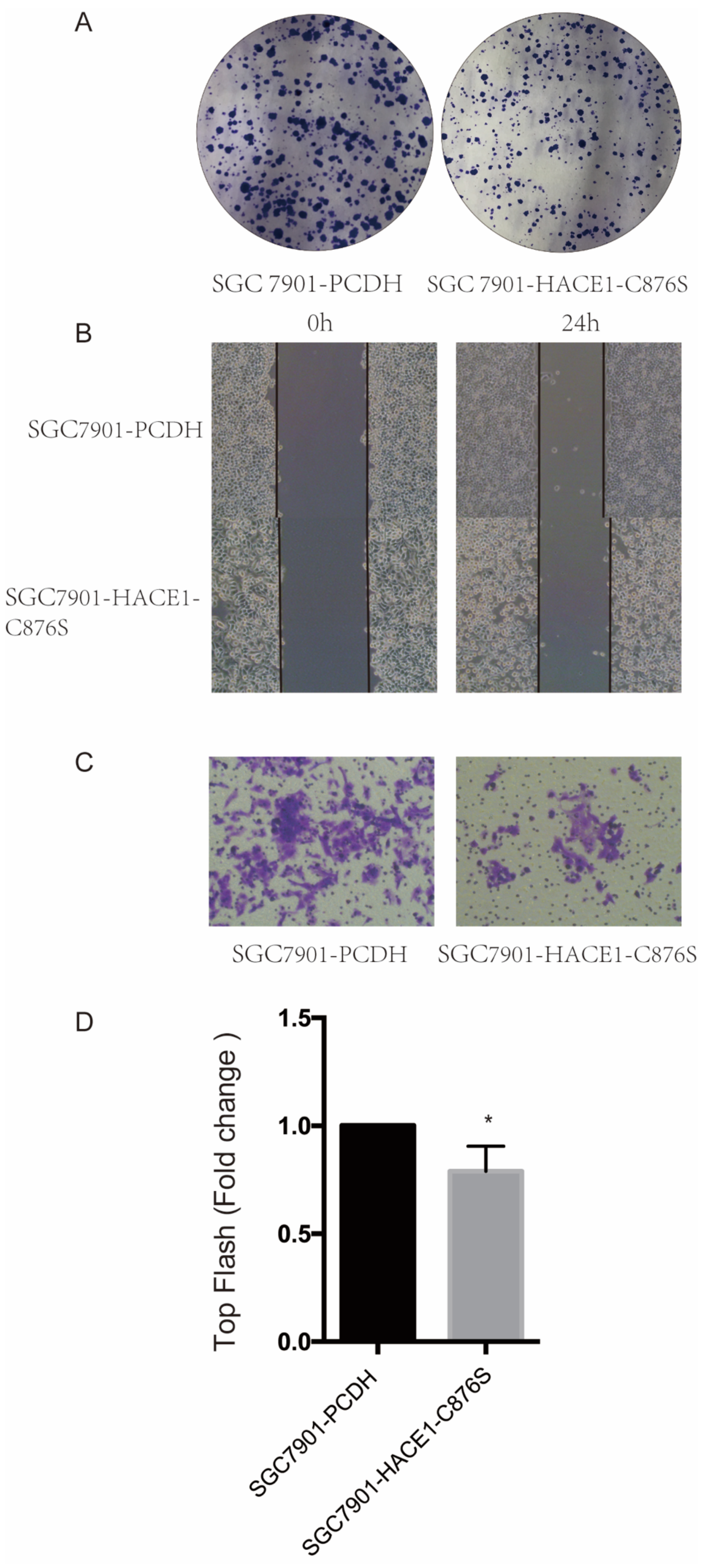
A
SGC 7901-PCDH
SGC 7901-HACE1-C876S
B
0h
24h
SGC7901-PCDH
SGC7901-HACE1-C876S
C
SGC7901-PCDH
SGC7901-HACE1-C876S
D
Top Flash (Fold change)
1.5
1.0
0.5
0.0
*
SGC7901-PCDH
SGC7901-HACE1-C876S

S1. The effect of HACE1 mutation on gastric cancer cell lines. (A) Pictures of colony formation of SGC7901 cell lines with or without overexpressing HACE1's mutant, HACE1-C876S. (B) Pictures of the wound-healing assay of SGC7901 with or without HACE1-C876S. These pictures were taken at 0 and 24 h, respectively (200×). (C) The images of migrating cells of SGC7901 with or without HACE1 mutation in Transwell assay. (D) The activity of the Wnt/β-catenin signaling pathway by TopFlash assay in SGC7901 with or without HACE1 mutation (*$P < 0.05$).

We established an inactive HACE1 mutant, SGC7901-HACE1-C876S, to explore whether HACE1 regulated tumor growth and migration through its E3 ligase activity. However, the results showed the same suppressive effect on cell proliferation (A) and migration (B,C) in gastric cancer cells as the wide type of HACE1 did. And this HACE1 mutation also inhibited the activity of the Wnt/β-catenin signaling pathway (D). Whether C876S did not work in cell lines or this mutation did not affect the function of HACE1 in gastric cancer remained unclear. To figure it out, we established a completely inactive mutant with HACE1's HECT domain deleted, HACE1-deltaHECT, and explored its effect on cell proliferation and migration. Those results could be found in Figure 6.
